# Author Correction: Identification of functional tetramolecular RNA G-quadruplexes derived from transfer RNAs

**DOI:** 10.1038/s41467-017-02140-9

**Published:** 2017-12-05

**Authors:** Shawn M. Lyons, Dorota Gudanis, Steven M. Coyne, Zofia Gdaniec, Pavel Ivanov

**Affiliations:** 1 Division of Rheumatology, Immunology and Allergy, Brigham and Women’s Hospital, Boston, MA 02115 USA; 2 Department of Medicine, Harvard Medical School, Boston, MA 02115 USA; 3 Institute of Bioorganic Chemistry, Polish Academy of Sciences, Noskowskiego 12/14, 61-704 Poznan, Poland; 4 The Broad Institute of Harvard and M.I.T., Cambridge, MA 02142 USA

*Nature Communications* **8**:1127 doi:10.1038/s41467-017-01278-w; Article published online: 24 October 2017

The original version of this Article contained an error in the spelling of the author Steven M. Coyne, which was incorrectly given as Stephen M. Coyne. This has now been corrected in both the PDF and HTML versions of the Article.

