# Supplementary material for: Identification of functional tetramolecular RNA G-quadruplexes derived from transfer RNAs
Source: Nat Commun. 2017 Oct 24;8:1127. doi: 10.1038/s41467-017-01278-w (PMC5655342; doi:10.1038/s41467-017-01278-w)
Supplement: Supplementary file 1 — Supplementary Information [file 41467_2017_1278_MOESM1_ESM.pdf]

Supplementary Figure 1

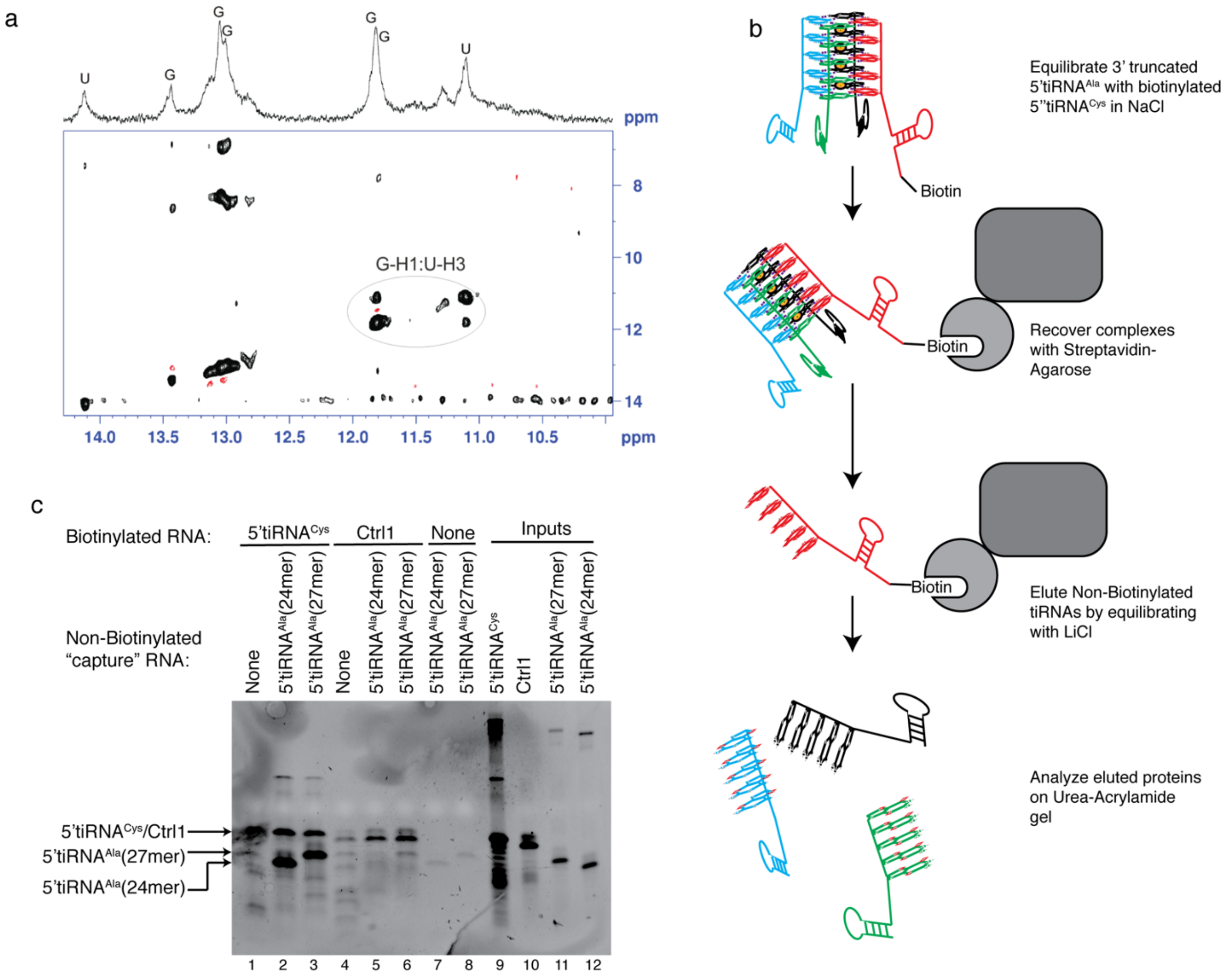

Supplementary Figure 1. (a) Figure S1. Imino-imino and imino-amino region of 2D NOESY spectrum (250 ms mixing time) of 5'tiRNA^{Ala} in the presence of 150 mM NaCl, 10 mM sodium phosphate and 0.1 mM EDTA, pH 6.8 at 10 °C. The assignment of resonances was obtained based on the observation of NOE cross-peaks characteristic of imino protons involved in G-C and A-U Watson-Crick base pairs formation. G-U base-pair was inferred from strong NOE between imino G-H1 and U-H3 hydrogens. (b) Heterogenous tetramer capture workflow. Biotinylated 5'tiRNA^{Cys} or Ctrl1 were equilibrated with unbiotinylated 5'tiRNA^{Ala}(24mer) or 5'tiRNA^{Ala}(27mer) in NaCl. Biotinylated complexes were recovered with streptavidin agarose and RG4 structures were disassembled by equilibrating in 150 mM LiCl. Eluted RNAs were analysed on acrylamide gels stained with Sybr Gold. (c) 5'tiRNA^{Cys} was able to associate and pull down both 5'tiRNA^{Ala}(24mer) and 5'tiRNA^{Ala}(27mer), while Ctrl1 RNA was not. Neither 5'tiRNA^{Ala} RNA was pulled down in the absence of biotinylated RNA (In 7 and 8).

Supplementary Figure 2

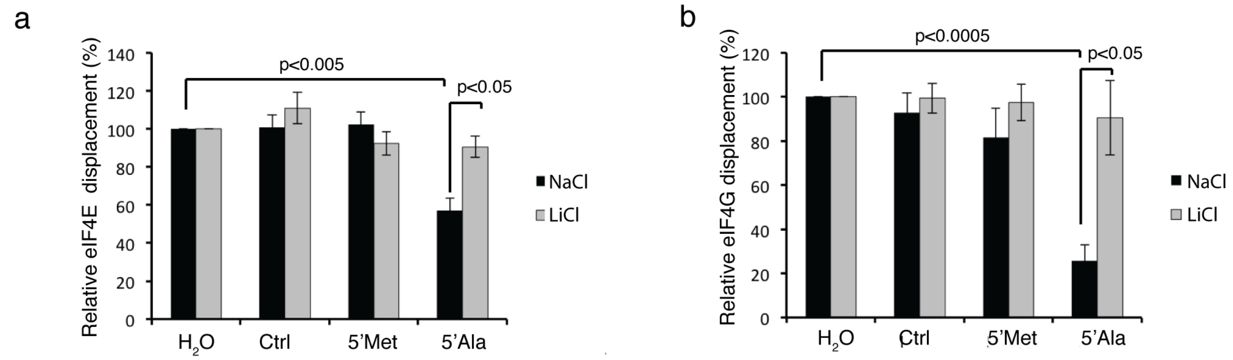

Supplementary Figure 2. (a) Quantification of eIF4E binding from figure 3d. (b) Quantification of eIF4G binding from figure 3d.

Supplemental Figure 3

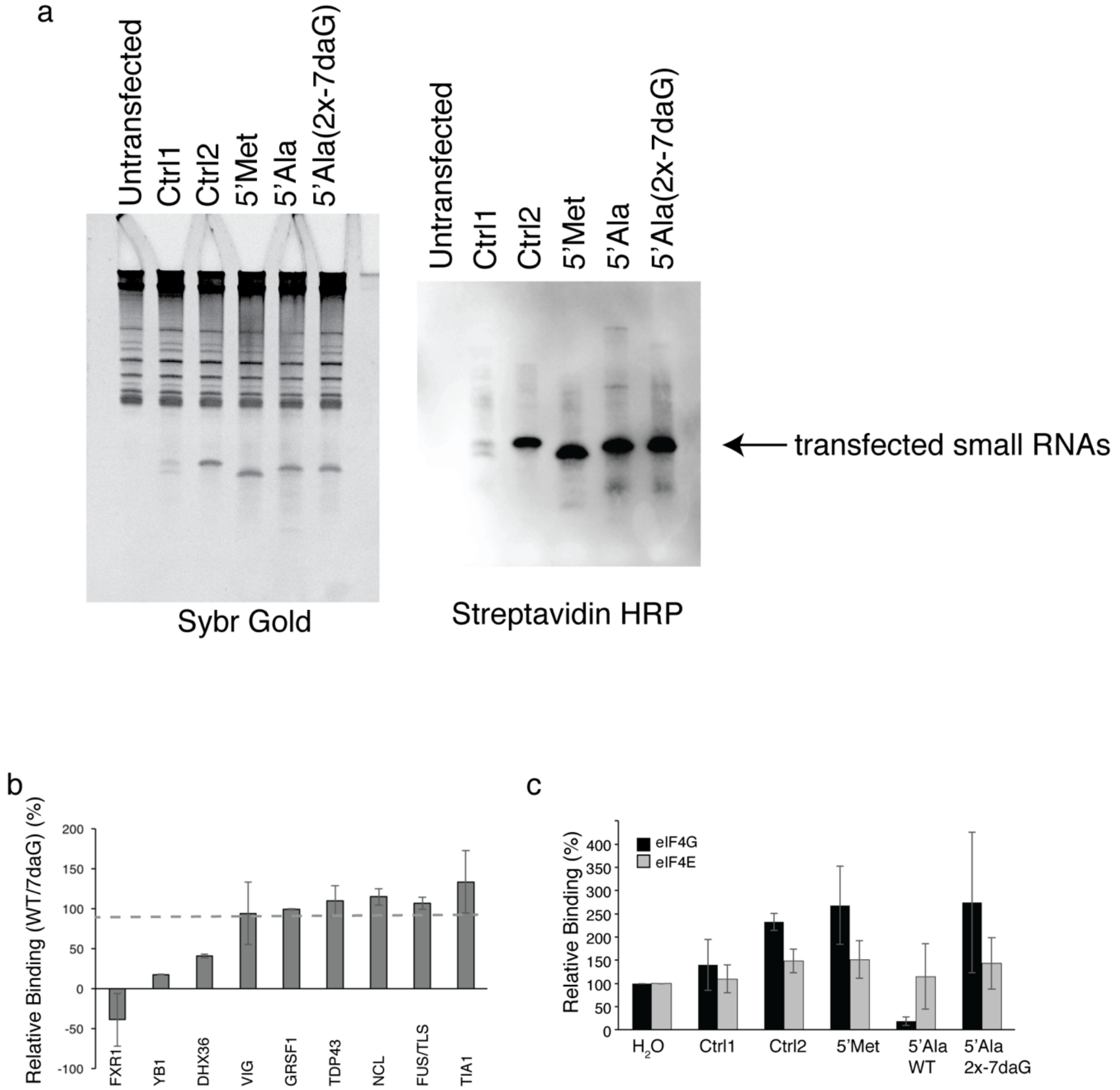

Supplementary Figure 3. (a) RNA was extracted from tiRNA transfected cells and run on Urea-acrylamide gel and stained with sybr gold (Left). RNA was transferred to nylon membrane and probed with Streptavidin-HRP to detect biotinylated tiRNAs (Right). There was no difference in the stability or transfection efficiency between 5'tiRNA^{Ala}(WT) and 5'tiRNA^{Ala}(2x-7daG) (b) Quantification of relative binding change from Figure 5c. (c) Quantification of eIF4E and eIF4G binding from figure 5d.

Supplementary Figure 4

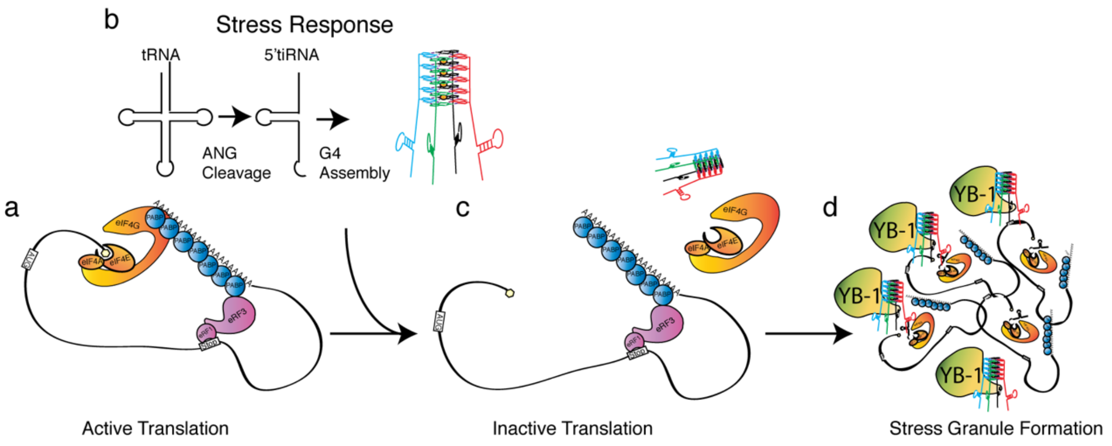

Supplementary Figure 4. Model of 5'tiRNA^{Ala} activity. (a) Active translation complexes in which polysomes are circularized through interaction with eIF4F (orange) and poly(A) binding protein (blue). (b) During stress response, cytoplasmic tRNAs are cleaved within their anticodon loop by ANG to produce tiRNAs. Following cleavage, 5'TOG containing tiRNAs assemble into tetramers coordinated by G-quadruplexes. (c) Tetramolecular 5'tiRNA^{Ala} displaces eIF4F from active translation complexes, thereby inhibiting translation. (d) Inactive translation complexes assemble into stress granules, mediated by YB-1.

Supplemental Figure 5

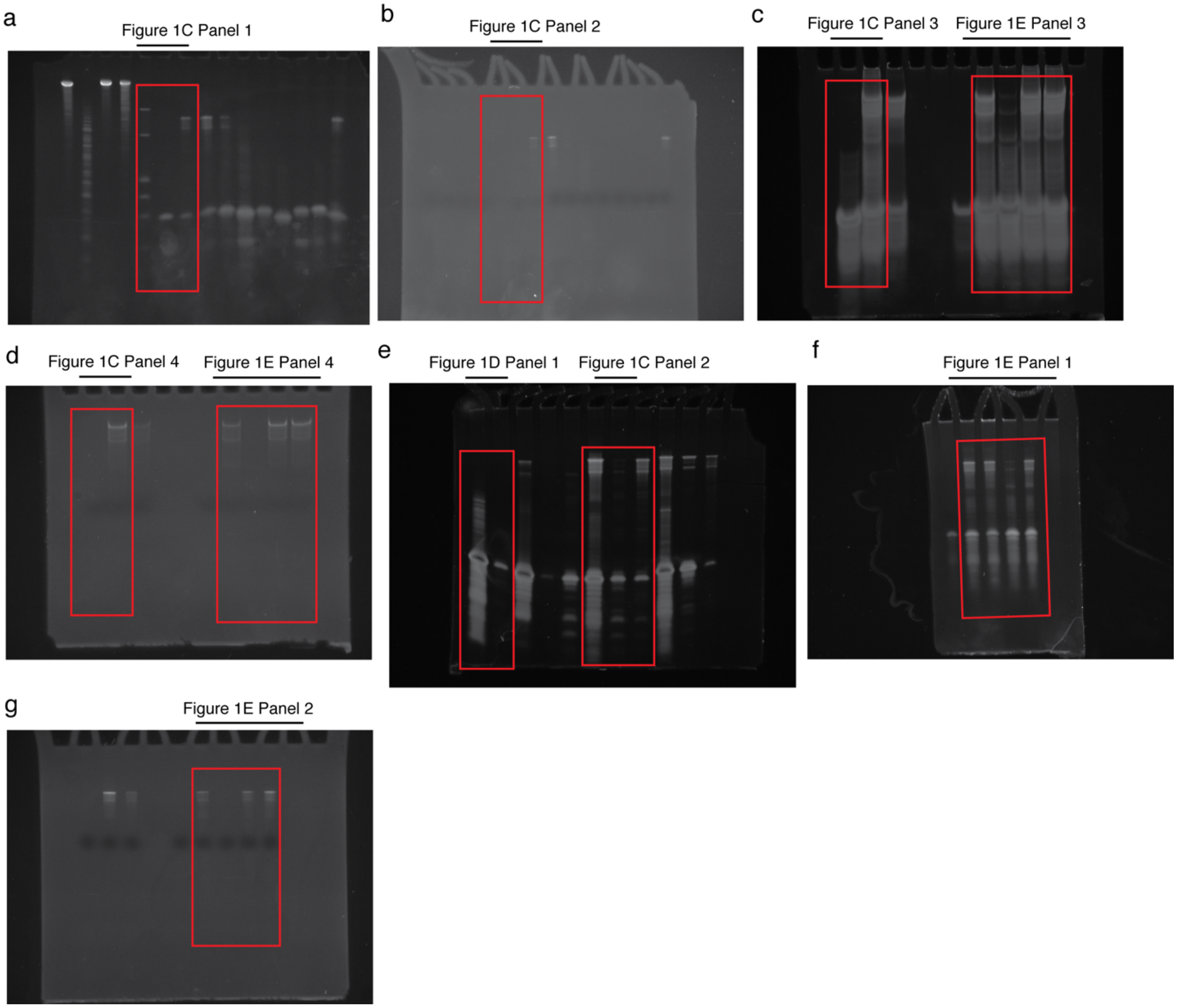

Supplementary Figure 5. Uncropped and unmodified data related to Figure 1. (a) Data for Figure 1C, panel 1 (b) Data for Figure 1C, Panel 2 (c) Data for Figure 1C, Panel 3 and Figure 1E, Panel 3, (d) Data for Figure 1C Panel 4 and Figure 1E, Panel 4, (e) Data for Figure 1D, (f) Data for Figure 1E Panel 1, (g) Data for Figure 1E Panel 2.

Supplementary Figure 6

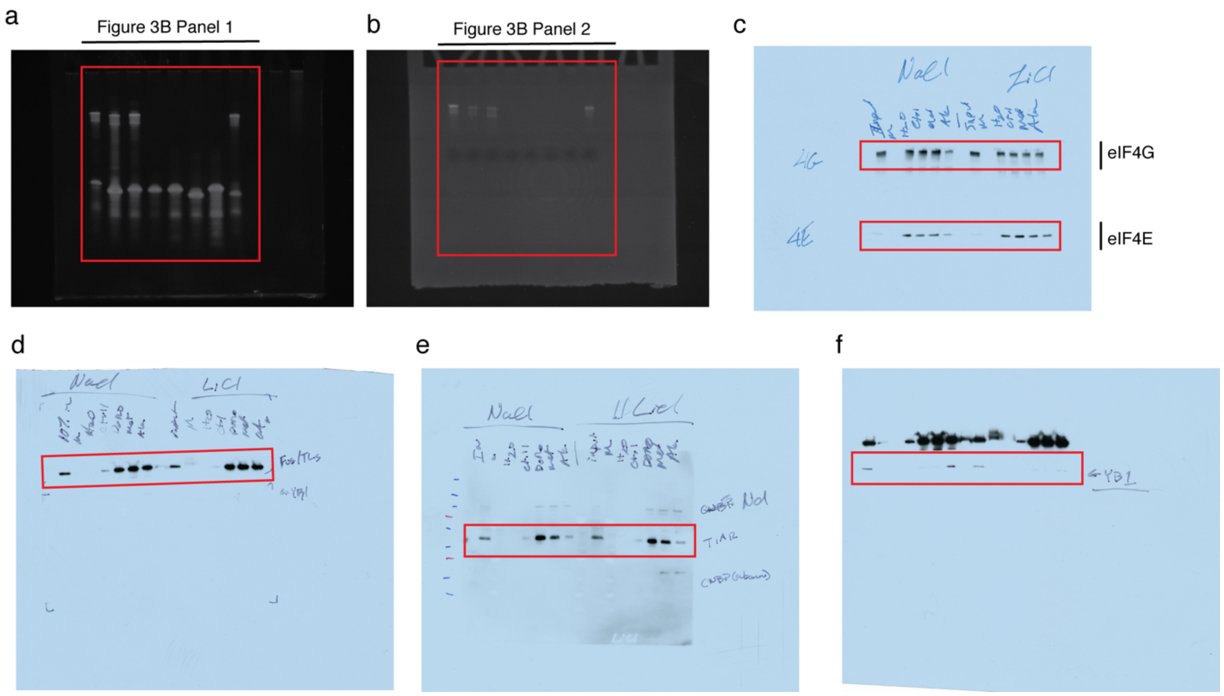

Supplementary Figure 6. Uncropped and unmodified data related to Figure 3. (a) Data for Figure 3b Panel 1, (b) Data for Figure 3b Panel 2 (c) Data for figure 3c. Western blots for eIF4G and eIF4E). (d) Data for Figure 3d. Western Blot for Fus/TLS. (e) Data for Figure 3e. Western Blot for TIAR. (f) Data for Figure 3e. Western blot for YB-1.

Supplementary Figure 7

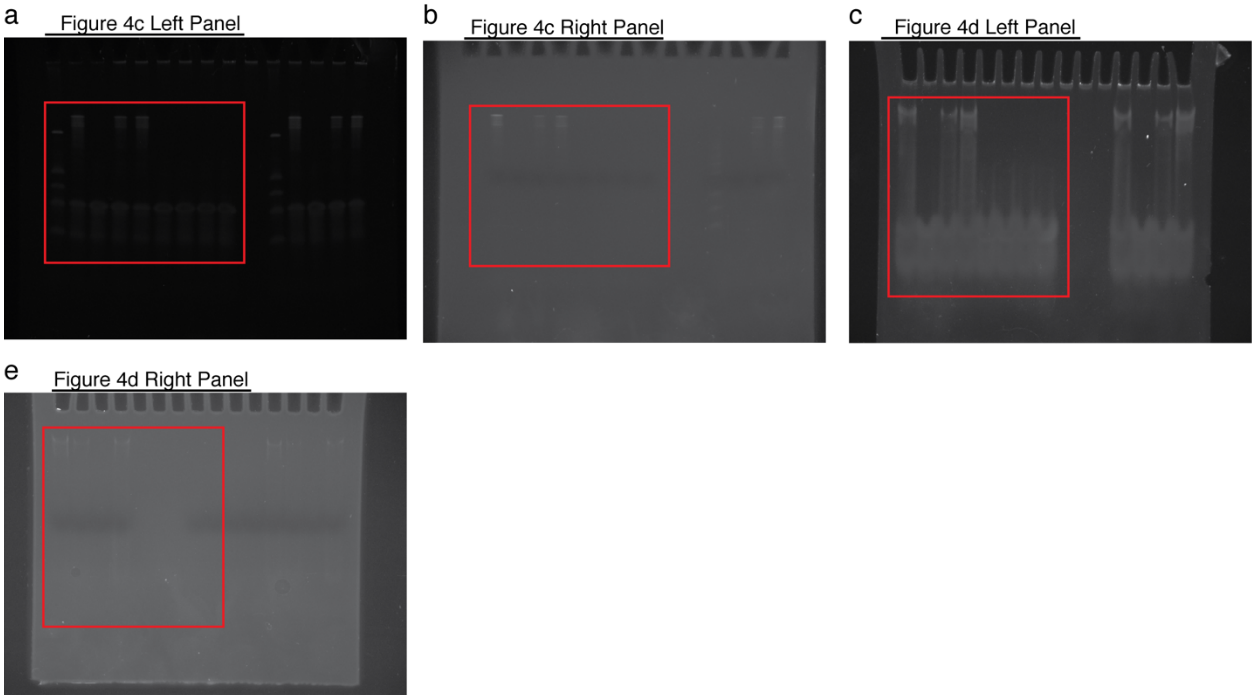

Supplementary Figure 7. Uncropped and unmodified data related to Figure 4. (a) Data for Figure 4c, Left Panel, (b) Data for Figure 4c, Right Panel, (c) Data for Figure 4d, Left Panel, (d) Data for Figure 4d, Right Panel.

Supplementary Figure 8

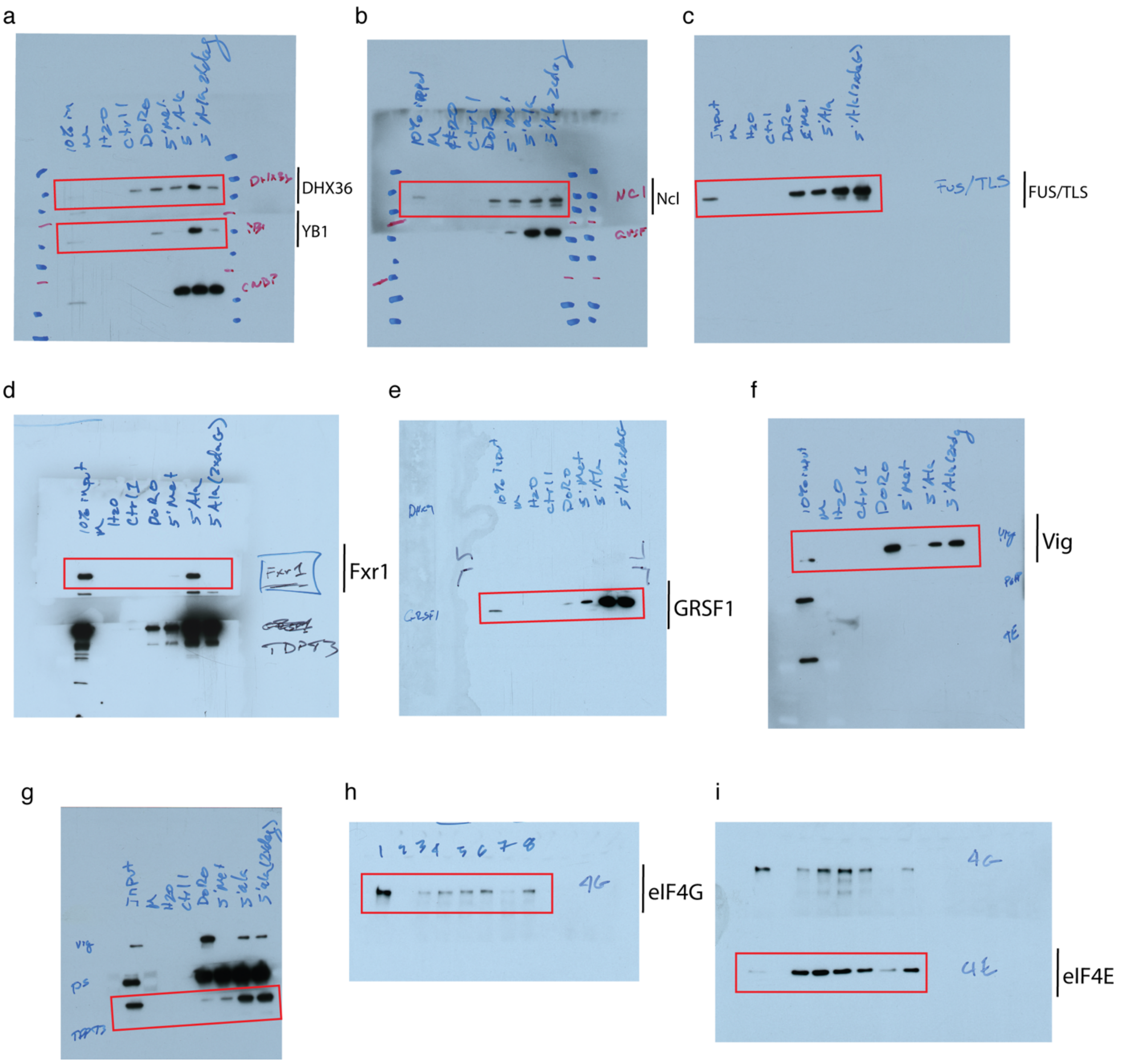

Supplementary Figure 8. Uncropped and unmodified data related to Figure 5. (a) Data for Figure 5c. Western blots for DHX36 and YB-1 (b) Data for Figure 5c. Western blot for Nucleolin (c) Data for Figure 5c. Western blot for FUS/TLS. (d) Data for Figure 5c. Western blot for Fxr1. (e) Data for Figure 5c. Western blot for GRSF1. (f) Data for Figure 5c. Western blot for Vigilin (g) Data for Figure 5c. Western blot for TDP43. (h) Data for Figure 5d. Western blot for eIF4G. (i) Data for Figure 5d. Western blot for eIF4E.

Supplementary Figure 9

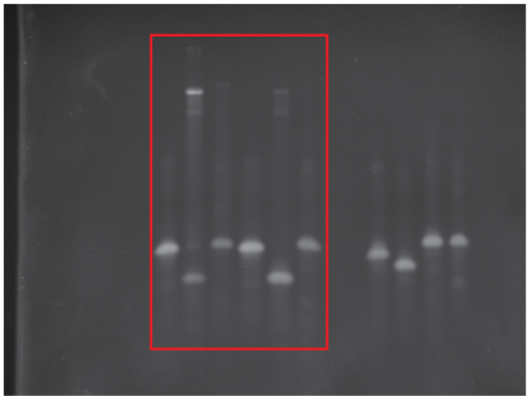

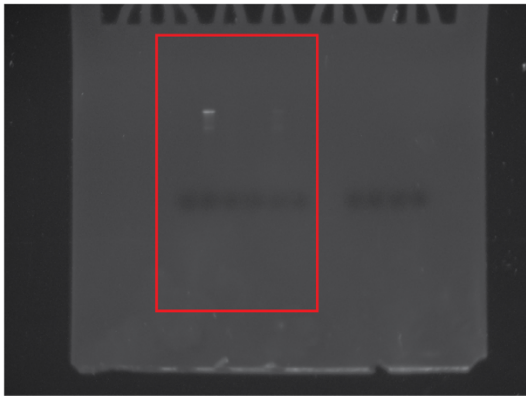

Supplementary Figure 9. Uncropped and unmodified data related to Figure 6. (a) Data for Figure 6b, Left Panel. (b) Data for Figure 6b, Right Panel

Supplementary Table 1

tRNA Gene	Sequence
tRNA-Asp-ATC-chr6-103	GGGGGTATAGCTCAGTGGTAGAGAGTGTACTTATCATGCACGAGGTCTTGGGCTGATTCCCCAGTACCTCCA
tRNA-Tyr-GTA-10-1	GGGGGTATAGCTCAGGGCTAGAGCTTTTTGACTGTAGAGCAAGAGGTCCCTGGTTCAAATCCAGGTTCTCCCT
tRNA-Val-AAC-6-1	GGGGGTGTAGCTCAGTGGTAGAGCGTATGCTTAACATTCATGAGGCTCTGGGTTCGATCCCCAGCACTTCCA

Supplementary Table 1. Identification of tRNA genes from which TOG-containing tiRNAs can be produced. TOG sequences highlighted in green.

Supplementary Table 2

Name	5'TOG Sequence	% SG	Relative Translation	eIF4F Displacement
None	----	0.9 ± 0.6	100	No
5'Ala	GGGGG	14.0 ± 2.3	9.6 ± 1.2	Yes
5'Ala(U4G)	UGGGG	8.9 ± 2.3	11.5 ± 1.9	Yes
5'Ala(4G)	GGGG	12.3 ± 1.7	22.8 ± 8.8	Yes
5'Ala(UU3G)	UUGGG	2.1 ± 1.1	79.8 ± 8.8	No
5'Ala(3G)	GGG	2.2 ± 0.7	81.7 ± 8.6	No
5'Met	----	1.2 ± 0.8	101.6 ± 16	No
5'Met(5G)	GGGGG	6.7 ± 1.8	15.6 ± 8.7	Yes

Adapted from Ivanov *et. al* (2011), Mol. Cell

Supplementary Table 2. Sequence of 5'TOG motifs mutations and correlation with previously reported biological activity.

Supplementary Table 3. Sequence of oligonucleotides used for this study.

Name	Sequence
Ctrl1	Phospho-UGAAGGGUUUUUUGUGUCUCUAUUUCCUUC-Biotin
Ctrl2	Phospho-GCAUUCACUUGGAUAGUAAAUCCAAGCUGAA-Biotin
5'tiRNA^{Ala}	Phospho-GGGGGUGUAGCUCAGUGGUAGAGCGCGUGC-Biotin
5'tiRNA^{Ala} (U4G)	Phospho-UGGGGUGUAGCUCAGUGGUAGAGCGCGUGC-Biotin
5'tiRNA^{Ala} (4G)	Phospho-GGGGUGUAGCUCAGUGGUAGAGCGCGUGC-Biotin
5'tiRNA^{Ala} (UU3G)	Phospho-UUGGGUGUAGCUCAGUGGUAGAGCGCGUGC-Biotin
5'tiRNA^{Ala} (3G)	Phospho-GGGUGUAGCUCAGUGGUAGAGCGCGUGC-Biotin
5'tiRNA^{Ala} (AA3G)	Phospho-AAGGGUGUAGCUCAGUGGUAGAGCGCGUGC-Biotin
5'tiRNA^{Met}	Phospho-GCCUCGUUAGCGCAGUAGGUAGCGCGUCAGU-Biotin
5'tiRNA^{Met} (3G)	Phospho-GGGGGUUAGCGCAGUAGGUAGCGCGUCAGU-Biotin
5'tiRNA^{Ala} (27mer)	Phospho-GGGGGUGUAGCUCAGUGGUAGAGCGCG
5'tiRNA^{Ala} (27mer)	Phospho-GGGGGUGUAGCUCAGUGGUAGAGC

Supplementary Table 4. Antibodies used in this study and concentrations

Protein	Company	Catalog #	Western blot dilution	Immunofluorescence Dilution
YB-1	AbCam	Ab12148	1:10,000	N/A
G3BP1	Santa Cruz	Sc81940	1:2,000	1:250
TIA-1	Santa Cruz	Sc-1751	1:1,000	N/A
TIAR	Santa Cruz	Sc-1749	1:1,000	1:250
eIF4G	Santa Cruz	Sc-11373	1:1,000	1:250
Nucleolin	Santa Cruz	Sc-9893	1:1,000	N/A
eIF4E	Santa Cruz	Sc-9976	1:1,000	N/A
FUS/TLS	Protein Tech Group	11570-1-AP	1:2,000	N/A
TDP-43	Protein Tech Group	10782-2-AP	1:2,000	N/A
Vigilin	Santa Cruz	2404C4a	1:1,000	N/A
DHX36	Protein Tech Group	13159-1-AP	1:2,000	N/A
GRSF1	Aviva	ARP40382-P050	1:2,000	N/A
